# Tin Complexes Containing an Atenolol Moiety as Photostabilizers for Poly(Vinyl Chloride)

**DOI:** 10.3390/polym12122923

**Published:** 2020-12-06

**Authors:** Baneen Salam, Gamal A. El-Hiti, Muna Bufaroosha, Dina S. Ahmed, Ahmed Ahmed, Mohammad Hayal Alotaibi, Emad Yousif

**Affiliations:** 1 Department of Chemistry, College of Science, Al-Nahrain University, Baghdad 64021, Iraq; baneenbano94@gmail.com; 2 Cornea Research Chair, Department of Optometry, College of Applied Medical Sciences, King Saud University, P.O. Box 10219, Riyadh 11433, Saudi Arabia; 3 Department of Chemistry, College of Science, United Arab Emirates University, P.O. Box 15551, Al-Ain 1818, UAE; muna.bufaroosha@uaeu.ac.ae; 4 Department of Medical Instrumentation Engineering, Al-Mansour University College, Baghdad 64021, Iraq; dinasaadi86@gmail.com; 5 Polymer Research Unit, College of Science, Al-Mustansiriyah University, Baghdad 10052, Iraq; drahmed625@gmail.com; 6 National Center for Petrochemicals Technology, King Abdulaziz City for Science and Technology, P.O. Box 6086, Riyadh 11442, Saudi Arabia

**Keywords:** poly (vinyl chloride), atenolol-tin complexes, photostabilization, weight loss, roughness factor, surface morphology

## Abstract

The lifetime of poly(vinyl chloride) (PVC) can be increased through the addition of additives to provide protection against irradiation. Therefore, several new tin complexes containing atenolol moieties were synthesized and their photostabilizing effect on PVC was investigated. Reacting atenolol with a number of tin reagents in boiling methanol provided high yields of tin complexes. PVC was then mixed with the tin complexes at a low concentration, producing polymeric thins films. The films were irradiated with ultraviolet light and the resulting damage was assessed using different analytical and surface morphology techniques. Infrared spectroscopy and weight loss determination indicated that the films incorporating tin complexes incurred less damage and less surface changes compared to the blank film. In particular, the triphenyltin complex was very effective in enhancing the photostability of PVC, and this is due to its high aromaticity (three phenyl rings) compared to other complexes. Such an additive acts as a hydrogen chloride scavenger, radical absorber, and hydroperoxide decomposer.

## 1. Introduction

Synthetic polymers such as polyester, polystyrene, polypropylene, polyethylene, and poly(vinyl chloride) (PVC) have many applications [1,2]. Plastics can substitute for construction materials such as wood, glass, and metals. The properties of plastics (e.g., their color, rigidity, toughness, transparency, and density) can be easily controlled during the manufacturing process [3]. PVC is a versatile and valuable thermoplastic that has unique strength and durability and is widely used in packaging, electronics, chemical engineering, architecture, and transportation applications [4,5]. It has good physical and mechanical stability, resists alkaline and acidic reagents, and is inexpensive to produce. However, environmental factors such as light, irradiation, and oxidants damage PVC and shorten its lifespan, especially when used outdoors. Therefore, the photostability of PVC should be enhanced to reduce the harmful effects of photooxidation and photodegradation.

PVC undergoes photooxidation upon exposure to ultraviolet (UV) irradiation, and the damage that takes place is due mainly to the autocatalytic dehydrochlorination process [6,7]. Eliminating hydrogen chloride (HCl) from PVC leads to the formation of polymeric stretches that contain conjugated polyene residues, cross-links, and chain-scissions [8]. These result in discoloration, cracks, decreases in overall weight and average molecular weight, loss of transparency, and changes in mechanical properties [9,10,11]. To enhance resistance of PVC against irradiation, light, and heat, UV absorbers, light screeners, free-radical scavengers, excited state energy quenchers, hydroperoxide decomposers, heat stabilizers, and HCl removers should be added to polymers [12].

PVC additives should be inexpensive to produce, and effective at producing the desired stabilizing effect at low concentrations [13,14]. Also, they should be stable, nonvolatile, nontoxic, nonhazardous, compatible with PVC, and they should not change the color of polymeric blends [13,14]. PVC industrial additives include *tris*(di-*tert*-butylphenyl)phosphite, *bis*(2-ethylhexyl)phthalate, 3,3′,4,4′-tetrachlorobiphenyl, and inorganics such as barium-zinc stabilizers [14]. These materials mainly act as PVC plasticizers, flame retardants, and antioxidants. However, the majority of these additives have been banned from use; in particular, they cannot be added to PVC that is used in medicinal applications because they are either carcinogenic, environmentally hazardous, or require co-stabilizers [15]. Therefore, new types of additives are needed to overcome the limitations of the currently used additives.

PVC additives should act as efficient primary and secondary stabilizers, thus, their structures should contain aromatic moieties and heteroatoms to efficiently absorb UV light and to form excited state complexes that are stabilized through resonance. A number of additives have been investigated for their protective effects on PVC films. The most recent studied and commonly used PVC additives include Schiff bases [16,17,18,19,20,21,22], aromatics containing heteroatoms [23,24,25,26], polyphosphates [27,28,29,30], tin complexes [31,32,33,34,35], titanium dioxide [36,37], nanoparticles [38,39], and others [40,41,42].

Organotin complexes are highly stable and act as efficient HCl scavengers. Atenolol is a stable, nontoxic, and inexpensive white solid that contains both an aryl ring (aromatic) and heteroatoms (nitrogen and oxygen). It is expected to act as a UV absorber, and when mixed with PVC, it produces neither a color change nor environmental problems. This paper discusses, for the first time, the synthesis and use of new tin complexes containing an atenolol unit and various substitutions as PVC photostabilizers.

## 2. Materials and Methods

### 2.1. General

PVC was obtained from Petkim Petrokimya (Istanbul, Turkey). The tin reagents; namely triphenyltin chloride (Ph_3_SnCl; 95%), dibutyltin oxide (Bu_2_SnO; 98%), dibutyltin dichloride (Bu_2_SnCl_2_; 96%), and dimethyltin dichloride (Me_2_SnCl_2_; 97%) were purchased from Merck (Gillingham, UK). The electronic spectra (200–900 nm) was measured in dimethylformamide (1 × 10^−3^ M) using a Shimadzu-160 spectrophotometer (Shimadzu, Kyoto, Japan). FTIR spectra (400–400 cm^−1^) using a KBr disc technique were recorded on a Shimadzu FTIR 8300 spectrophotometer (Shimadzu, Tokyo, Japan). ^1^H (500 MHz) and ^13^C NMR (125 MHz) spectra were recorded in DMSO-*d*_6_ using a Bruker DRX500 NMR spectrometer (Bruker, Zürich, Switzerland). Energy dispersive X-ray (EDX) images were captured using a Bruker XFlash 6 10 (Bruker, Tokyo, Japan) and the microscopic images were captured by a Meiji Techno Microscope (Meiji Techno, Tokyo, Japan). The field emission scanning electron microscope (FESEM) images (15 kV) were captured by a TESCAN-MIRA3 system (TESCAN, Kohoutovice, Czech Republic). A Veeco atomic force microscope (AFM) (Veeco Instruments Inc., Plainview, NY, USA) was used to capture AFM images. An accelerated weather-meter QUV tester (Q-Panel Company; Homestead, FL, USA) was used to irradiate PVC films using UV light (λ_max_ = 365 nm; light intensity = 6.43 × 10^−9^ ein dm^−3^s^−1^) at 25 °C.

### 2.2. Synthesis of Complexes **1**–**4**

**Scheme 1:**
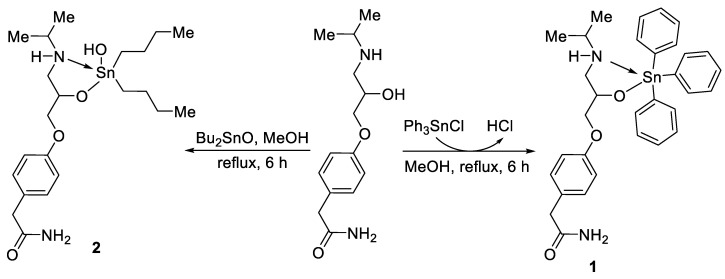
Synthesis of complexes **1** and **2**.

**Scheme 2:**
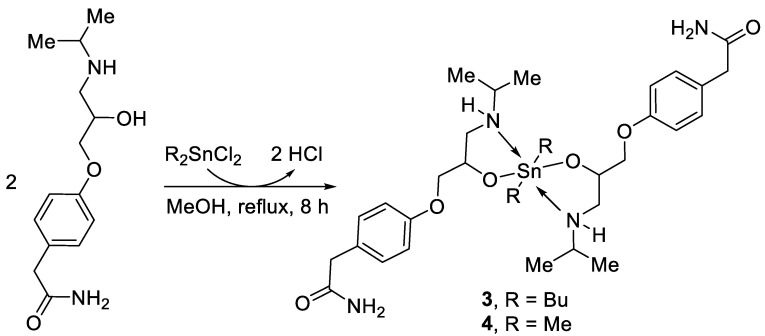
Synthesis of complexes **3** and **4**.

Atenolol (2.0 mmol) and a tin reagent (1.1 or 2.2 mmol) were mixed together in MeOH (30 mL) and then refluxed for 6–8 h (Scheme 1 and Scheme 2). The mixture was left overnight and the solid that was formed was collected by filtration, washed with water, and recrystallized using MeOH to give the corresponding tin complex.

### 2.3. Preparation of PVC Films

Each tin complex (25 mg) was added to a PVC (5.0 g) solution in tetrahydrofuran (THF; 100 mL) to produce a homogenous mixture. The mixture was stirred for 20 min at 25 °C and transferred into a clean glass plate containing 15 holes 40 μm deep. The plate was left at 25 °C for 24 h and the resulting films were dried in a vacuum oven at 40 °C for 6 h.

## 3. Results and Discussion

### 3.1. Synthesis of Complexes **1**–**4**

The atenolol-tin complexes **1**–**4** (Scheme 1 and Scheme 2) were produced in 69%–84% yields in the form of white solids (Table 1). The 6-h reactions between atenolol and triphenyltin chloride or dibutyltin oxide (1:1 molar ratio) in dry MeOH yielded tin complex **1** or **2**, respectively (Scheme 1). Similarly, reacting atenolol (two mole equivalents) with dibutyltin dichloride or dimethyltin dichloride yielded either complex **3** or **4**, respectively (Scheme 2). The yields were relatively low for complexes **2** and **3**, but no side-products were observed, although some materials were lost in the crystallization processes.

The structures of tin complexes **1–4** were elucidated based on the spectral data obtained from the UV, FTIR, and NMR spectroscopy along with the elemental composition analysis (Table 1, Table 2, Table 3 and Table 4). The electronic spectra of complexes **1–4** (Figures S1–S4) mainly show the presence of two absorptions bands corresponding to the π–π* (225–275 nm) and n–π* (291–431 nm).

The FTIR spectra of complexes **1–4** (Figures S5–S8) confirm the absence of the stretching vibrational band for the hydroxyl group, which appeared at 3356 cm^−1^ for the ligand (e.g., atenolol). The stretching vibrational band corresponding to the secondary NH bond in complexes **1–4** was slightly shifted to either lower (e.g., complexes **3** and **4**) or higher (e.g., complex **1**) frequencies compared to that for the ligand. This clearly indicates that the NH group takes part in the coordination with the tin atom [42]. The FTIR spectra of complexes **1–4** showed absorption bands corresponding to the Sn–C, Sn–O, and Sn–N groups that appeared at the 563–570 cm^−1^, 501–543 cm^−1^, and 447–478 cm^−1^ regions, respectively [43]. The asymmetric vibration bands for the carbonyl group in complexes **1–4** appeared as very strong signals at the 1639–1674 cm^−1^ region. The difference between the wavenumbers (ν) of the asymmetric (asym) and symmetric (sym) vibrational bands for the absorption of carbonyl groups confirms the bidentate interaction between oxygen and tin atoms in complexes **1–4** [44]. The most common FTIR absorption bands for these complexes are shown in Table 2.

The ^1^H NMR spectra of complexes **1–4** show the presence of aryl protons as two doublets (*J* = 7.8**–**8.0 HZ) appearing at the 7.19**–**6.52 ppm region. They also show exchangeable signals that correspond to the NH and NH_2_ protons (Table 3). The CH**–**O and CH_2_**–**O protons appear as broad peaks at the 4.15**–**3.48 ppm and 3.94**–**3.41 ppm regions, respectively. The spectra also show the presence of other protons at the expected chemical shifts (ppm). The ^1^H NMR spectrum of **1** is shown in Figure S9.

The ^13^C NMR spectra of complexes **1–4** provide further confirmation for their structures (Table 4). The carbonyl group carbons and the C-1 carbon of the aryl group attached to the oxygen atom appears downfield at the 172.1–172.6 ppm and 156.6–157.3 ppm regions, respectively. The carbons of the CH–O and CH_2_–O groups appear at regions 69.6–70.7 ppm and 65.5–68.1 ppm, respectively. The ^13^C NMR spectra of complexes **1–4** show all other carbons at the expected chemical shifts (ppm). The ^13^C NMR spectrum of **1** is shown in Figure S10.

**Figure 1:**
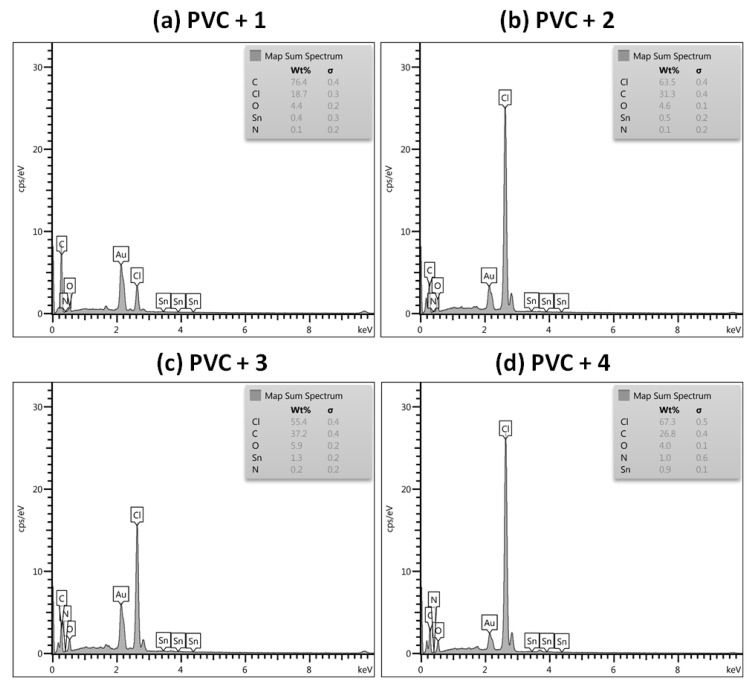
Energy-Dispersive X-Ray (EDX) patterns for poly(vinyl chloride) (PVC) films: (**a**) PVC + **1**, (**b**) PVC + **2**, (**c**) PVC + **3**, and (**d**) PVC + **4**.

Complexes **1**–**4** were added to PVC at a concentration of 0.5 wt% to produce colorless films. Previous studies suggest that this concentration provides the most desirable protection for the PVC blends [32,33]. The EDX patterns was used to identify the elements present in each tin complex. Each peak corresponds to a specific element and its height determines the relative atomic abundance. The EDX spectra of PVC films [45] show clear and highly abundant bands for the chlorine atom along with low intensity bands for tin, nitrogen, and oxygen atoms in PVC films incorporating complexes **1**–**4** (Figure 1). The bands assignment was in agreement with those previously reported for related elements [22,27,34].

**Figure 2:**
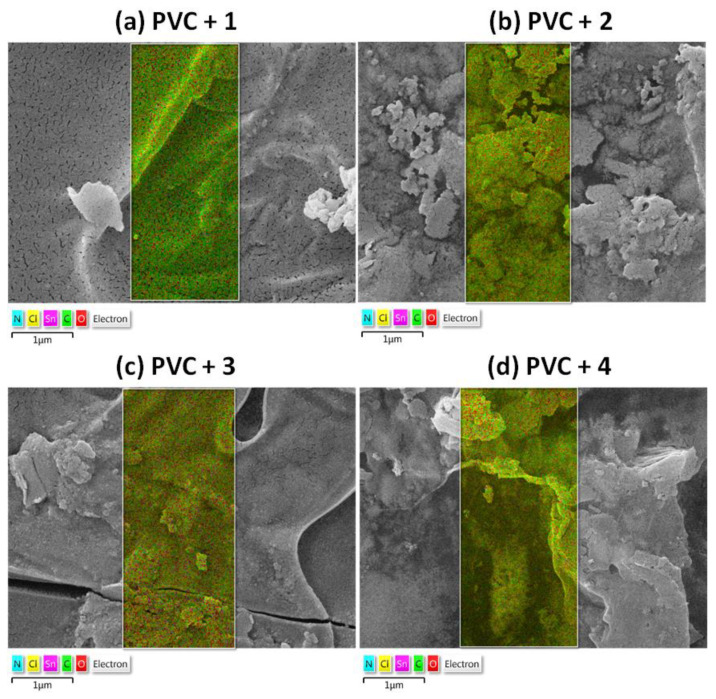
EDX mapping for PVC films: (**a**) PVC + **1**, (**b**) PVC + **2**, (**c**) PVC + **3**, and (**d**) PVC + **4**.

The EDX mapping of PVC films containing additives show the distribution and relative abundance of elements within the blends (Figure 2) [46]. Clearly, the images indicate the homogenous distribution of the elements from both tin complexes and PVC. Chlorine and carbon atoms are highly abundant and distributed thought the blends. In addition, the images indicate the presence of other elements (tin, nitrogen, and oxygen) within the complexes.

### 3.2. FTIR Spectroscopy

**Figure 3:**
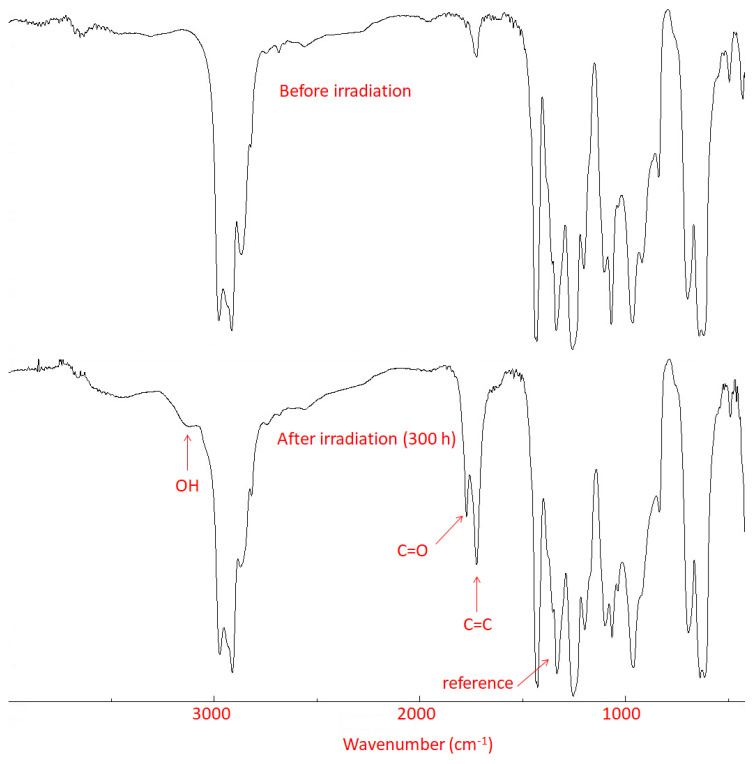
Fourier-transform infrared (FTIR) spectra for PVC (blank) film.

The photostability of PVC is poor in the 253–310 nm region, and the role of additives is to protect PVC by absorbing the UV light, thus reducing the rate of photooxidation and photodegradation. These processes drastically change the physical properties of PVC, producing discoloration, darkness, surface cracking, and softening. In addition, photooxidation changes various chemical properties of PVC. For example, it leads to the formation of fragments that contain–OH (hydroxyl; e.g., alcohols), C=O (carbonyl; e.g., ketones and acids), and C=C (alkene; e.g., unsaturated fragments) functional groups [47]. FTIR spectroscopy was performed to test the photochemical activities of complexes **1**–**4** as PVC additives. The PVC films were irradiated with UV light and the FTIR spectra were recorded after 50–300 h of irradiation. The spectra of PVC (blank) at 0 and 300 h of irradiation are shown in Figure 3. The FTIR spectrum of the irradiated PVC film containing complex **1** is shown in Figure S11. During irradiation (every 50 h), the growths in the absorbance of peaks corresponding to the–OH (3500 cm^−1^), C=O (1772 cm^−1^), and C=C (1604 cm^−1^) groups (*A*s) were measured and compared (using Equation (1)) with that of a standard peak (*A*r; C–H bonds) that appears at 1328 cm^−1^ [12,48].

(1) $$I_{s}\;=\;A_{s}/A_{r}$$

**Figure 4:**
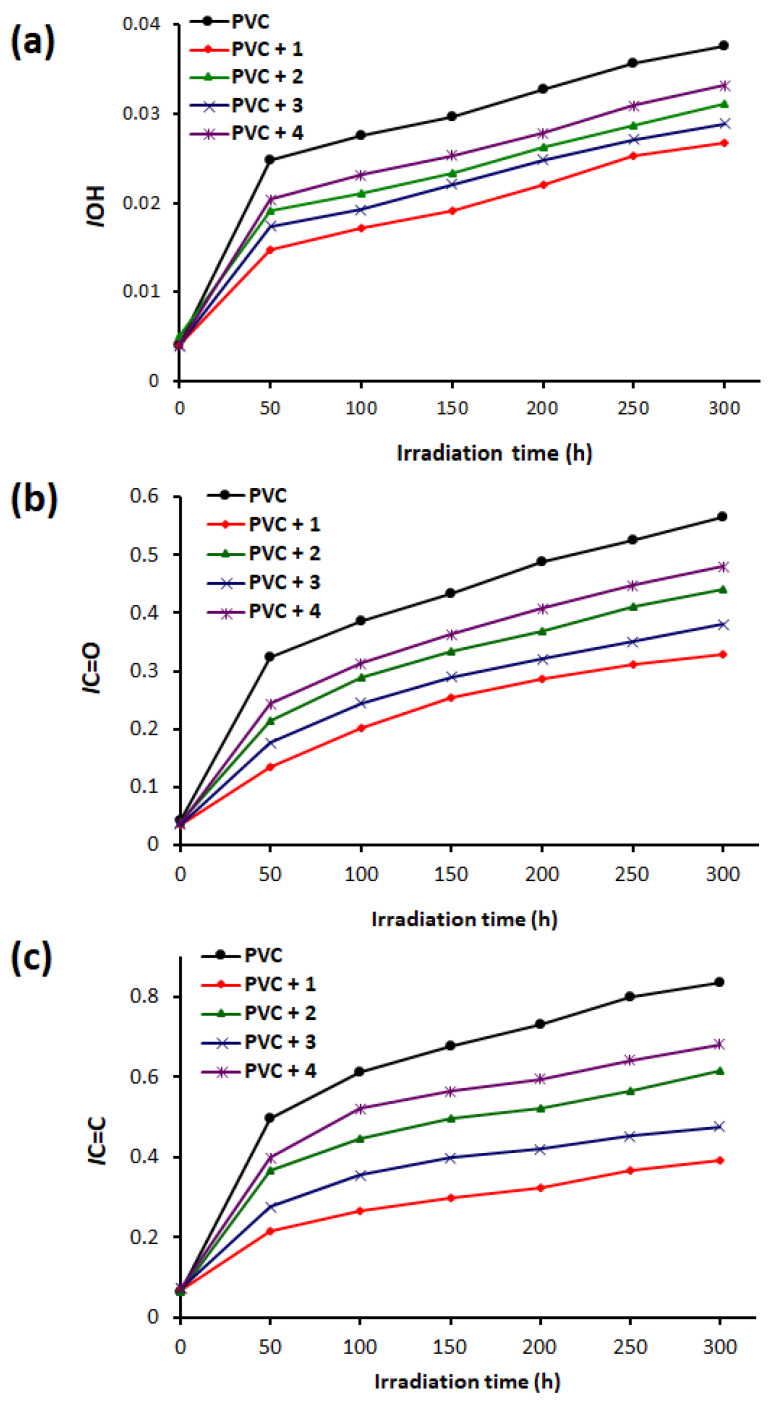
Changes in (**a**): *I*OH, (**b**): *I*C=O, and (**c**): *I*C=C for PVC films.

The changes in *I*s (*I*OH, *I*C=O, and *I*C=C) were higher for the blank PVC compared to those for the films containing complexes **1**–**4** (0.5 wt %) (Figure 4). The changes in *I*s occurred very rapidly in the first 50 h of irradiation and increased steadily thereafter. The *I*OH values (Figure 4a) for the blank PVC film was 0.025 and 0.038 after 50 and 300 h of irradiation, respectively. At the end of irradiation process, the *I*OH values were 0.027, 0.031, 0.028, and 0.033 for PVC + complex **1**, PVC + complex **2**, PVC + complex **3**, and PVC + complex **4**, respectively. Thus, in terms of efficiency as PVC stabilizers, the complexes performed in the order complex **1** > complex **3** > complex **2** > complex **4**. Complex **1,** which contains three phenyl groups (highly aromatic) in addition to an aryl moiety in the skeleton of atenolol, provides the highest level of PVC protection against photodegradation. Similar observations are apparent for *I*C=O and *I*C=C (Figure 4b,c). For example, the *I*C=O and *I*C=C values for the blank PVC film were 0.57 and 0.83, respectively, after 300 h of irradiation. On the other hand, the *I*C=O and *I*C=C values for the PVC + complex **1** film were much lower (0.33 and 0.39, respectively) at the end of the irradiation process (300 h).

### 3.3. Weight Loss

Photodegradation of PVC causes its dehydrochlorination and weight loss [12]. The percentage of PVC weight loss after irradiation can be used as an indicator of the performance of complexes **1**–**4** as photostabilizers. Therefore, the weight losses of the films after every 50 h of irradiation were calculated from the difference between PVC weight before and after irradiation (*W_0_* and *W_t_*, respectively), as shown in Equation (2) [17].

(2) $$Weight\;loss\;\left(\%\right)\;=\;\left[\left(W_{0}-W_{t}\right)/W_{0}\right]\;\times \;100$$

**Figure 5:**
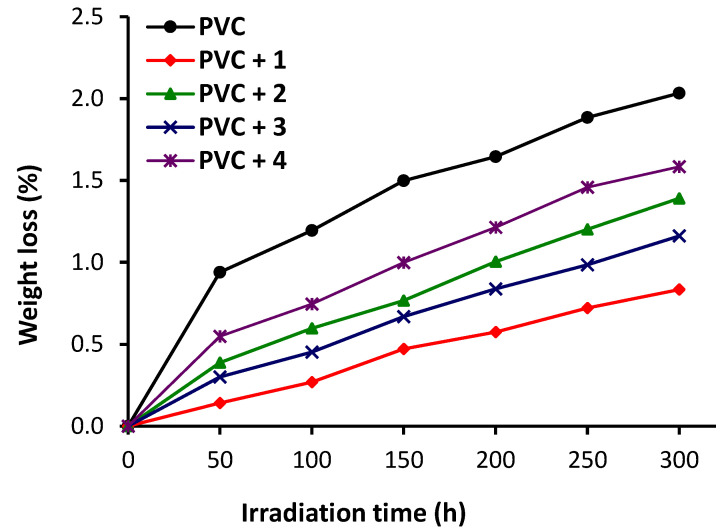
Changes in weight loss (%) for PVC films.

Figure 5 shows the changes in weight loss (%) for the PVC blends as a result of irradiation. Complexes **1**–**4** clearly reduce in the weight loss undergone by PVC in response to irradiation. The weight losses (%) relative to PVC (blank) of PVC + **1**, PVC + **2**, PVC + **3**, and PVC + **4** were 2.03, 0.83, 1.39, 1.16, and 1.58, respectively. Clearly, complex **1** provides the highest level of PVC protection.

### 3.4. Surface Morphology

**Figure 6:**
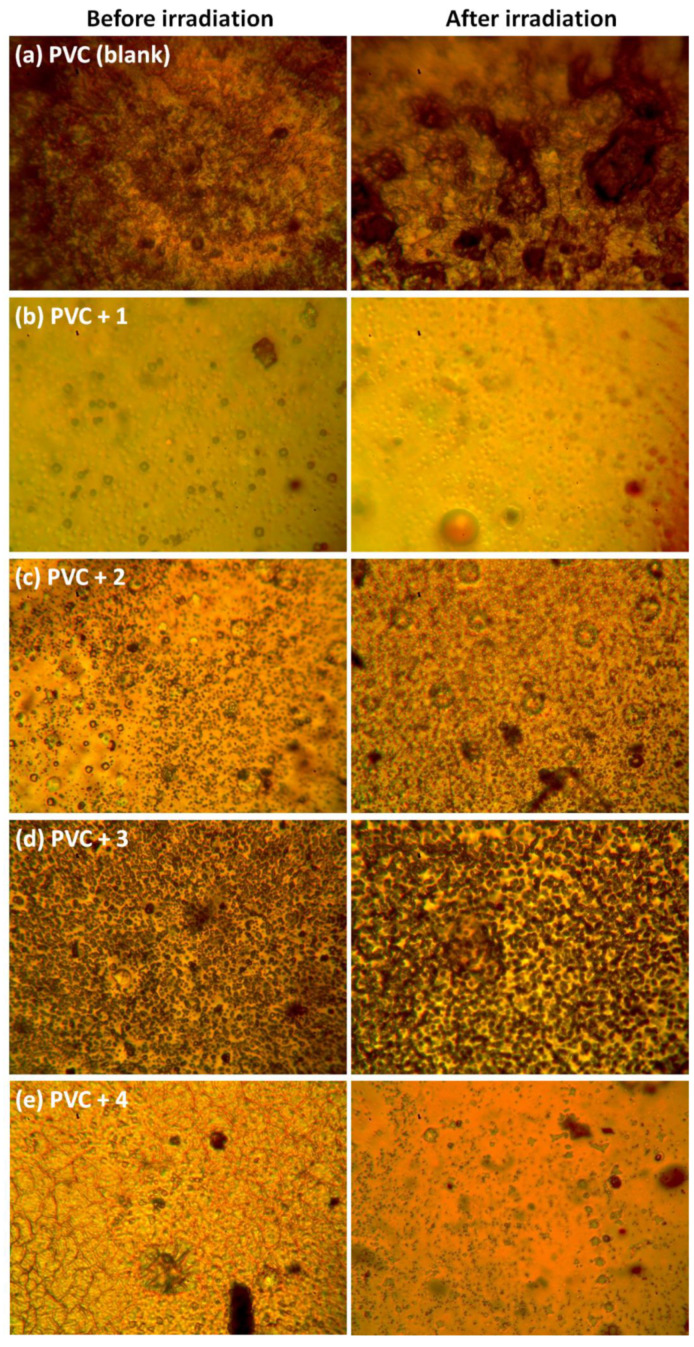
Optical images for PVC films (left) before and (right) after irradiation (300 h) at 400× magnification: (**a**) PVC (blank), (**b**) PVC + **1**, (**c**) PVC + **2**, (**d**) PVC + **3**, and (**e**) PVC + **4**.

Optical microscopy detects defects such as damages, irregularities, chain-scissions, and cracks on the surface of PVC after irradiation [49,50]. Moreover, optical microscopy provides information about the degree of crystallinity and roughness of PVC. Figure 6 shows that after irradiation, the surfaces of the films were rougher and contained large numbers of cracks, spots, and grooves compared to unirradiated films. These surface defects are mainly due to the elimination of HCl and the formation of small, unsaturated residues. The levels of damage to the PVC surface were generally lower in PVCs containing complexes **1**–**4**. In particular, after irradiation, the surface of the PVC film containing complex **1** was smoother and clearer, with fewer spots and cracks compared to the surfaces of films containing complexes **2**–**4**.

**Figure 7:**
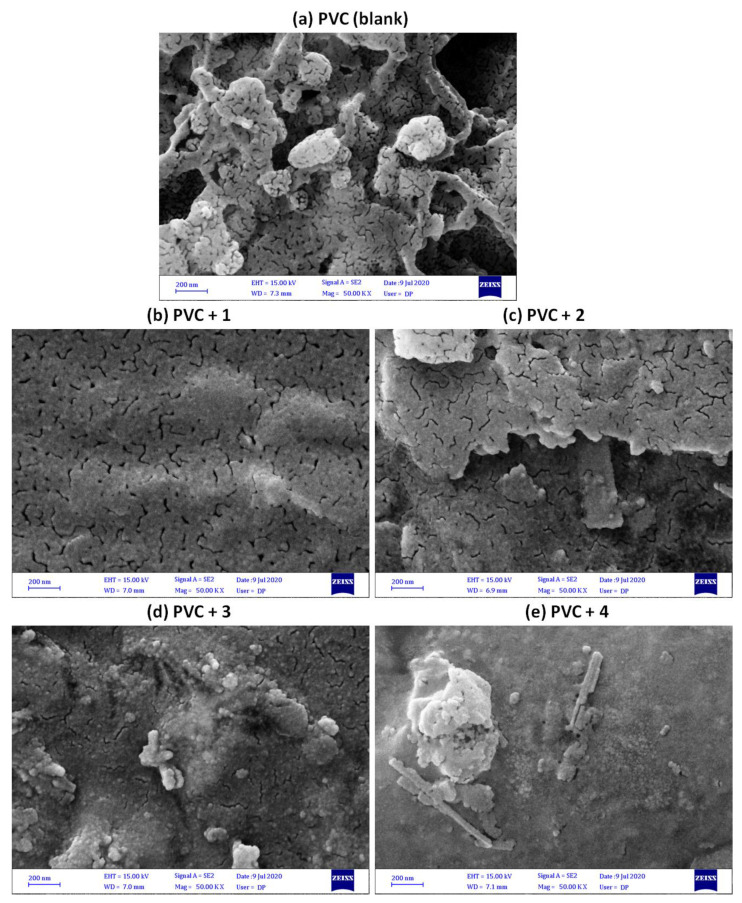
FESEM images for PVC films after irradiation (300 h): (**a**) PVC (blank), (**b**) PVC + **1**, (**c**) PVC + **2**, (**d**) PVC + **3**, and (**e**) PVC + **4**.

FESEM analysis provides information on particles shape and size, level of cross-sectioning, and homogeneity of PVC surfaces [51,52]. Before irradiation, FESEM images show that PVC films are highly homogeneous, with fairly smooth surfaces [30,31], and after irradiation, Figure 7 shows rough surfaces with cracking due to broken bonds and the elimination of HCl [53]. The roughness, heterogeneity, and long and deep cracks were very noticeable for the irradiated PVC (blank). In contrast, after irradiating PVCs containing additives **1**–**4,** the surfaces were smoother with less cracking compared to the blank PVC. In particular, the surface of the film containing complex **1** was almost clear, homogeneous, and smooth, appearing similar to a non-irradiated PVC. The results clearly show that complexes **1**–**4** act as PVC stabilizers.

**Figure 8:**
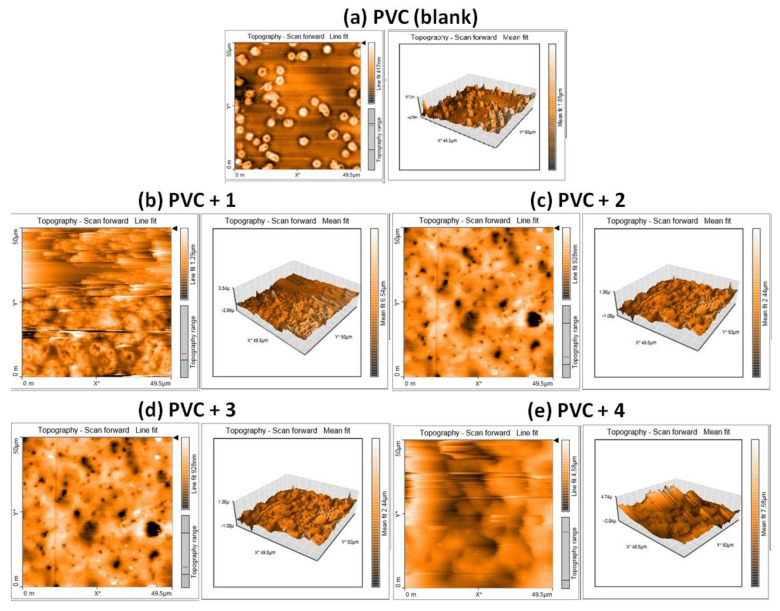
AFM images for PVC films after irradiation (300 h): (**a**) PVC (blank), (**b**) PVC + **1**, (**c**) PVC + **2**, (**d**) PVC + **3**, and (**e**) PVC + **4**.

The level of roughness of the surface of irradiated PVC films was measured using AFM, resulting in estimates of the roughness factor (*R*q), a measure of the degree of photodegradation due to the elimination of volatiles in response to irradiation [54]. The two- and three-dimensional AFM images of irradiated PVC films are shown in Figure 8 and their *R*q values are reported in Table 5. In the presence of additives, the PVC surface was smoother and regular compared to the blank film. The PVC blends incorporating complexes **1**–**4** had lower *R*q values than that for the blank film. Complex **1** improved the roughness factor by more than 7-fold, thus acting as an efficient PVC photostabilizer. The other complexes also provided reasonable degrees (at least 4-fold compared with the blank PVC film) of improvement in surface roughness. The performance levels of complexes **1**–**4** are better than many Schiff bases [18,19,22] and tin complexes containing 2-(4-isobutylphenyl) propanoate [41], naproxen [34], and furosemide [32] moieties; these complexes are comparable to tin complexes containing valsartan [31] and polyphosphates containing diethylenetriamine [27]; however, they perform less effectively compared to polyphosphates and tin complexes with high levels of aromaticity [28,41].

### 3.5. Photostabilization Mechanisms

**Figure 9:**
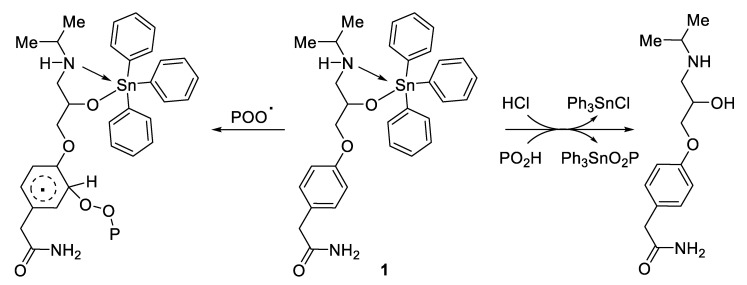
Complex **1** acts as a hydrogen chloride scavenger, hydroperoxide decomposer, and radical absorber.

Complexes **1**–**4** reduced the level of damage against PVC due to photoirradiation, and are thus considered PVC photostabilizers. The additives contain aromatic moieties that are cable of absorbing harmful irradiation (primary stabilizers via resonance) followed by the harmless release of energy over a long period of time [23]. The tin atom is highly acidic (Lewis acid) and therefore can efficiently scavenge HCl released during the PVC dehydrochlorination process. In addition, complexes **1**–**4** (in particular triphenyltin complex **1** (Figure 9)) acted as hydroperoxide decomposers with the release of free ligands (i.e., atenolol) [41]. Moreover, these additives can form stable complexes with chromophores (e.g., POO**^.^**) that transfer and release energy without damaging the polymeric materials (i.e., radical absorbers) [12]. The coordination between the polarized atoms within the PVC and atenolol can provide a secondary stabilization effect. Clearly, complexes **1**–**4** are able to provide PVC films with long-term protection against irradiation damage [25].

## 4. Conclusions

Here, we describe the synthesis of satisfactory levels of new tin complexes containing atenolol. The structures of the tin complexes were established using analytical and spectroscopic techniques. As additives to PVC, the atenolol-tin complexes provided significant photostabilization to the polymeric films. Analysis of surface morphology via infrared spectroscopy and weight loss studies prove that the triphenyltin complex is the most effective additive in terms of enhancing the photostability of the blends. This additive reduced the weight loss of PVC films by 2.4-fold and inhibited photodegradation by 1.4–2.1-fold. In addition, the triphenyltin complex improved the roughness factor of the irradiated materials by 7.5-fold. The additives acted as light absorbers, hydrogen chloride scavengers, hydroperoxide decomposers, and radical quenchers.

## Figures and Tables

**Table 1:** Melting point, yield, and elemental analyses for complexes **1–4**.

Complex	Melting Point (°C)	Yield (%)	Measured % (Calculated; %)		
			C	H	N
**1**	174–176	84	62.52(62.46)	5.97 (5.90)	4.50 (4.55)
**2**	223–225	70	51.36 (51.28)	7.94 (7.82)	5.35 (5.44)
**3**	208–211	69	56.69 (56.62)	7.99 (7.92)	7.25 (7.34)
**4**	198–200	76	53.15 (53.03)	7.20 (7.12)	8.18 (8.25)

**Table 2:** Common FTIR absorption bands for complexes **1–4**.

Complex	Wavenumber (ν; cm−1)							
	OH	NH_2_	C=O			Sn–C	Sn–O	Sn–N
			asym	sym	∆v (asym − sym)			
**1**	3375	3178	1639	1512	127	563	536	447
**2**	3356	3167	1674	1512	162	567	543	478
**3**	3332	3178	1670	1512	158	570	528	447
**4**	3332	3174	1639	1516	123	570	501	482

**Table 3:** ^1^H NMR data (500 MHz) for complexes **1–4**.

Complex	^1^H NMR (δ, ppm, *J* in Hz)
**1**	7.89–7.82 (m, 6H, Ar), 7.49–7.41 (m, 9H, Ar), 7.19 (d, *J* = 7.9 Hz, 2H, Ar), 6.89 (d, *J* = 7.9 Hz, 2H, Ar), 6.85 (s, exch., 2H, NH_2_), 3.94 (br, 1H, CH–O), 3.87 (br, 2H, CH_2_–O), 3.31 (s, 2H, CH_2_C=O), 2.79–2.72 (m, 2H, CH and NH), 2.61 (m, 2H, CH_2_), 1.02 (d, *J* = 7.5 Hz, 6H, 2Me)
**2**	6.72 (d, *J* = 7.8 Hz, 2H, Ar), 6.52 (d, *J* = 7.8 Hz, 2H, Ar), 6.36 (s, exch., 2H, NH_2_), 4.49 (s, exch., 1H, NH), 3.49 (br, 1H, CH–O), 3.41 (br, 2H, CH_2_–O), 2.85 (s, 2H, CH_2_C=O), 2.29–2.11 (m, 3H, CH_2_ and CH), 1.30–1.22 (m, 12H, 6 CH_2_), 1.06 (d, *J* = 7.5 Hz, 6H, 2 Me), 0.54 (t, *J* = 7.5 Hz, 6H, 2 Me)
**3**	7.05 (s, exch., 4H, 2 NH_2_), 6.75 (d, *J* = 7.8 Hz, 4H, Ar), 6.45 (d, *J* = 7.8 Hz, 4H, Ar), 3.72 (br, 2H, 2 CH–O), 3.51 (br, 4H, 2 CH_2_–O), 2.87 (s, 4H, 2 CH_2_C=O), 2.75–2.59 (m, 4H, 2 CH and 2 NH), 2.43 (m, 4H, 2 CH_2_), 1.27–1.21 (m, 12H, 6 CH_2_), 0.99 (d, *J* = 7.6 Hz, 12H, 4 Me), 0.44 (t, *J* = 7.6 Hz, 6H, 2 Me)
**4**	7.46 (s, exch., 4H, 2 NH_2_), 7.18 (d, *J* = 8.0 Hz, 4H, Ar), 6.87 (d, *J* = 8.0 Hz, 4H, Ar), 4.15 (br, 2H, 2 CH–O), 3.94 (br, 4H, 2 CH_2_–O), 3.21 (s, 4H, 2 CH_2_C=O), 3.05–3.01 (m, 4H, 2 CH and 2 NH), 2.87 (m, 4H, 2 CH_2_), 1.21 (d, *J* = 7.7 Hz, 12H, 4 Me), 0.72 (s, 6H, 2 Me)

**Table 4:** ^13^C NMR data (125 MHz) for complexes **1–4**.

Complex	^13^C NMR (δ, ppm)
**1**	172.5, 157.3, 136.0, 130.0, 128.9, 128.6, 128.4, 128.0, 114.2, 70.7, 68.1, 49.7, 48.3, 41.3, 22.5
**2**	172.1, 156.9, 129.5, 128.0, 113.8, 70.4, 68.0, 49.6, 47.7, 40.9, 40.1, 32.5, 27.8, 22.5, 13.7
**3**	172.1, 156.6, 129.6, 128.3, 113.8, 69.6, 65.5, 49.0, 47.2, 40.9, 27.4, 26.8, 25.4, 19.2, 13.2
**4**	172.6, 157.0, 130.0, 128.8, 114.3, 70.0, 65.8, 49.5, 47.4, 41.3, 19.4, 5.7

**Table 5:** *R*q values for PVC films after irradiation.

PVC Film	*R*q	Reduction in *R*q (fold)
PVC	322	—
PVC + **1**	43	7.5
PVC + **2**	75	4.3
PVC + **3**	70	4.6
PVC + **4**	81	4.0

## Supplementary Materials

The following are available online at https://www.mdpi.com/2073-4360/12/12/2923/s1, Figure S1: Electronic spectrum of complex 1, Figure S2: Electronic spectrum of complex 2, Figure S3: Electronic spectrum of complex 3, Figure S4: Electronic spectrum of complex 4; Figure S5: FTIR spectrum of complex 1, Figure S6: FTIR spectrum of complex 2, Figure S7: FTIR spectrum of complex 3, Figure S8: FTIR spectrum of complex 4, Figure S9: ^1^H NMR spectrum of complex 1, Figure S10: ^13^C NMR spectrum of complex 1, Figure S11: FTIR spectrum of PVC containing complex 1 after irradiation (300 h).
