# Supplementary material for: Tin Complexes Containing an Atenolol Moiety as Photostabilizers for Poly(Vinyl Chloride)
Source: Polymers (Basel). 2020 Dec 6;12(12):2923. doi: 10.3390/polym12122923 (PMC7768508; doi:10.3390/polym12122923)
Supplement: Supplementary file 1 [file polymers-12-02923-s001.zip › polymers-1002343-supplementary.docx]

Supplementary Material

**Tin Complexes Containing an Atenolol Moiety as Photostabilizers for Poly(Vinyl Chloride)**

**Baneen Salam ^1^, Gamal A. El-Hiti ^2,^*, Muna Bufaroosha ^3^, Dina S. Ahmed ^4^, Ahmed Ahmed ^5^, Mohammad Hayal Alotaibi ^6,^* and Emad Yousif ^1,^***

^1^ Department of Chemistry, College of Science, Al-Nahrain University, Baghdad 64021, Iraq; baneenbano94@gmail.com

^2^ Cornea Research Chair, Department of Optometry, College of Applied Medical Sciences,
King Saud University, P.O. Box 10219, Riyadh 11433, Saudi Arabia

^3^ Department of Chemistry, College of Science, United Arab Emirates University, P.O. Box 15551,
Al-Ain 1818, UAE; muna.bufaroosha@uaeu.ac.ae

^4^ Department of Medical Instrumentation Engineering, Al-Mansour University College, Baghdad 64021, Iraq; dinasaadi86@gmail.com

^5^ Polymer Research Unit, College of Science, Al-Mustansiriyah University, Baghdad 10052, Iraq; drahmed625@gmail.com

^6^ National Center for Petrochemicals Technology, King Abdulaziz City for Science and Technology,
P.O. Box 6086, Riyadh 11442, Saudi Arabia

***** Correspondence: gelhiti@ksu.edu.sa (G.A.E.-H.); mhhalotaibi@kacst.edu.sa (M.H.A.); emad_yousif@hotmail.com (E.Y.); Tel.: +966-11469-3778 (G.A.E.-H.); Fax: +966-11469-3536 (G.A.E.-H.)


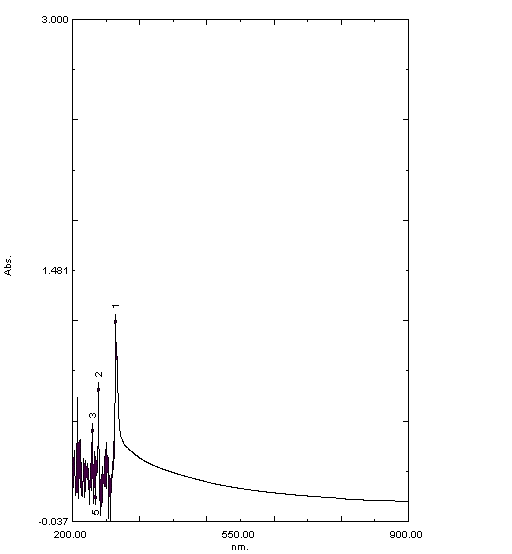


**Figure S1.** Electronic spectrum of complex **1**.


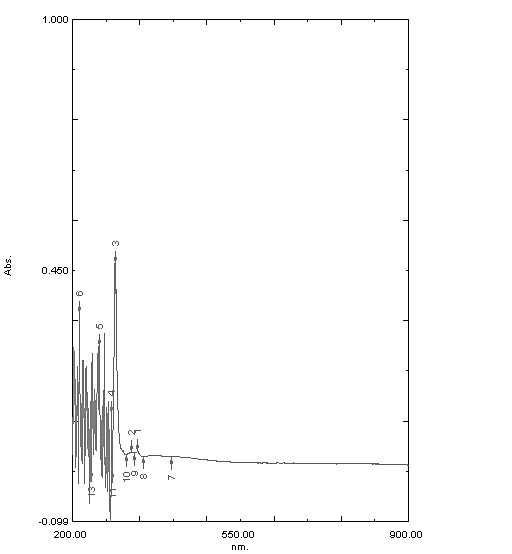


**Figure S2.** Electronic spectrum of complex **2**.


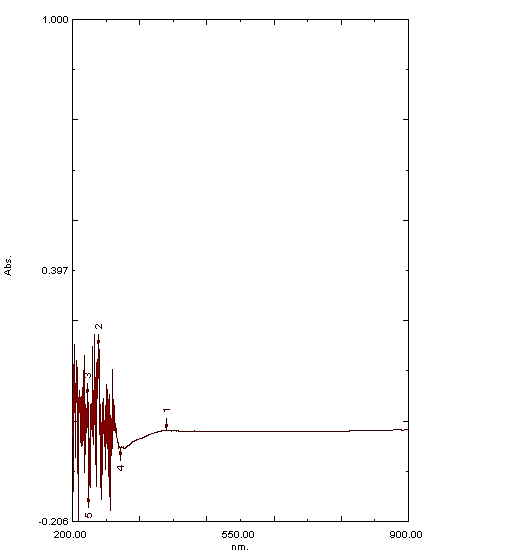


**Figure S3.** Electronic spectrum of complex **3**.


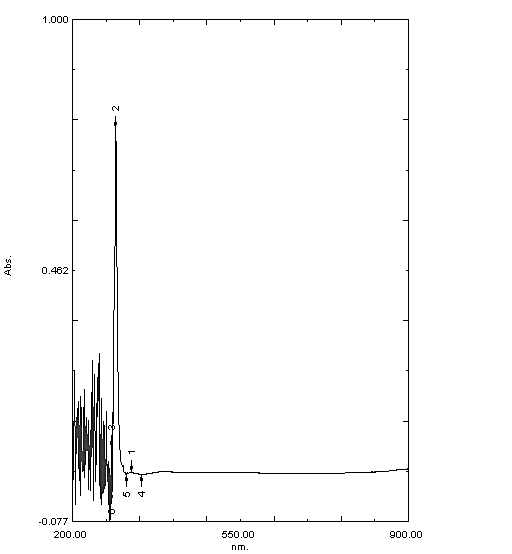


**Figure S4.** Electronic spectrum of complex **4**.


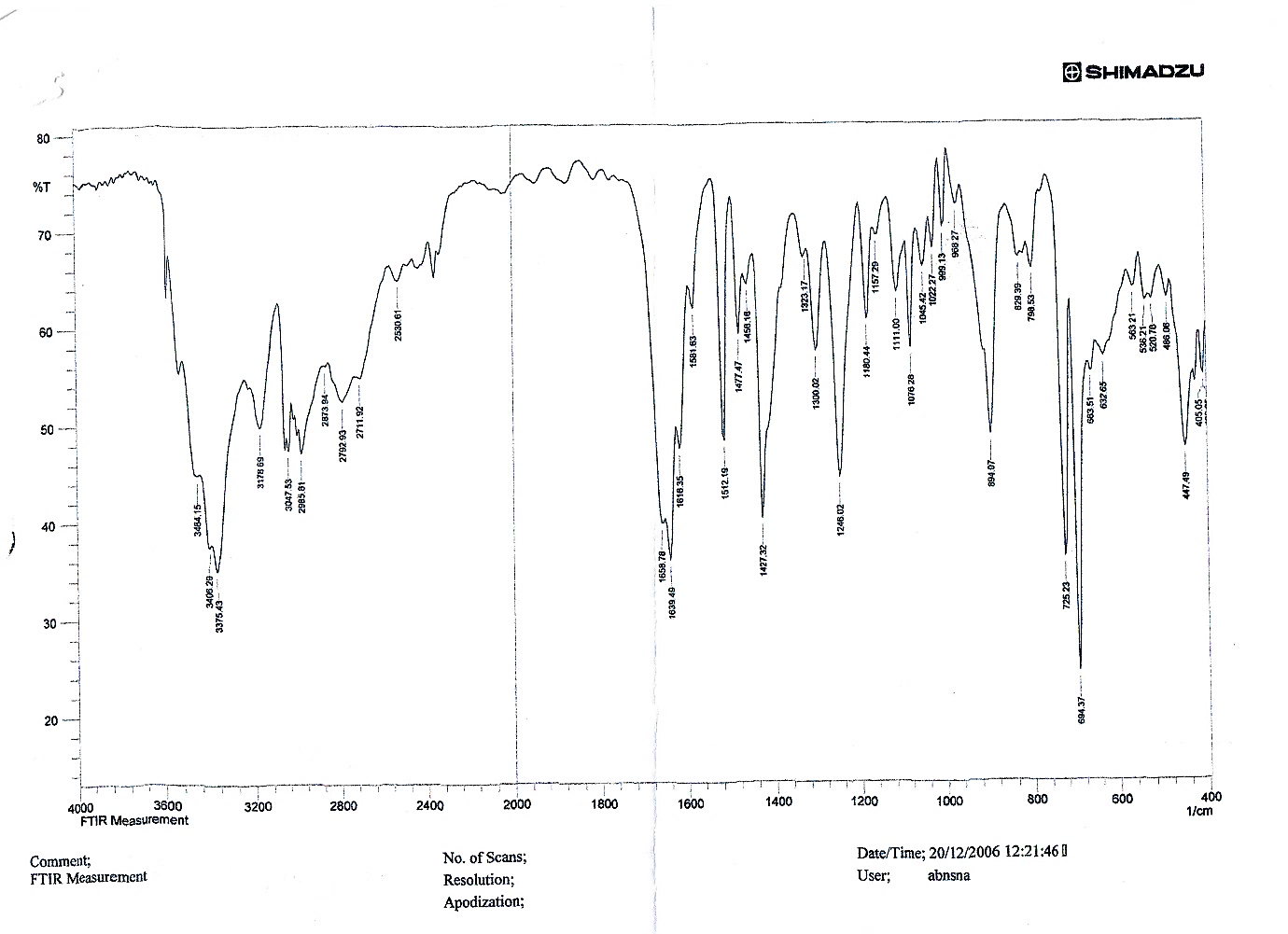


**Figure S5.** FTIR spectrum of complex **1**.


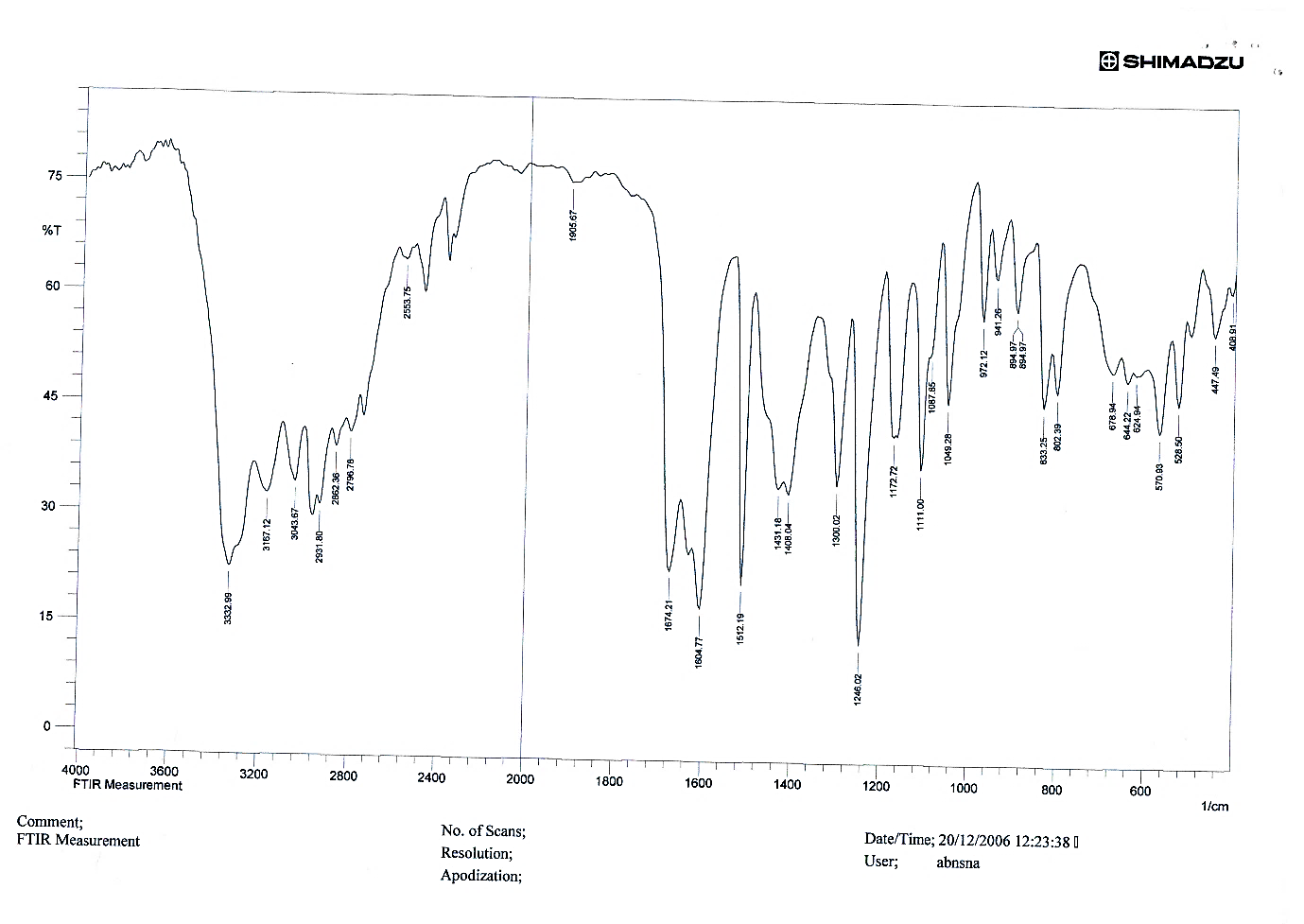


**Figure S6.** FTIR spectrum of complex **2**.


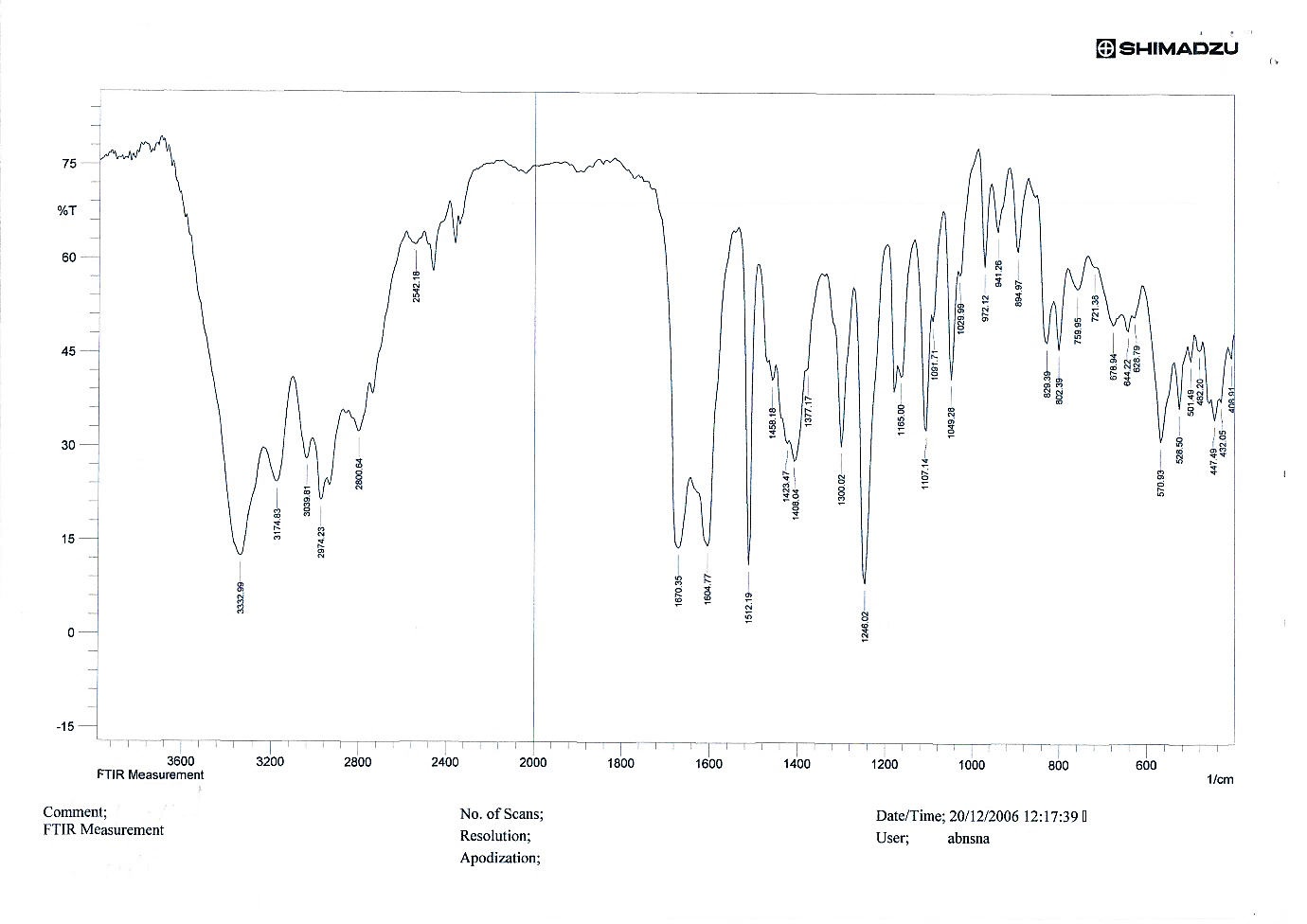


**Figure S7.** FTIR spectrum of complex **3**.


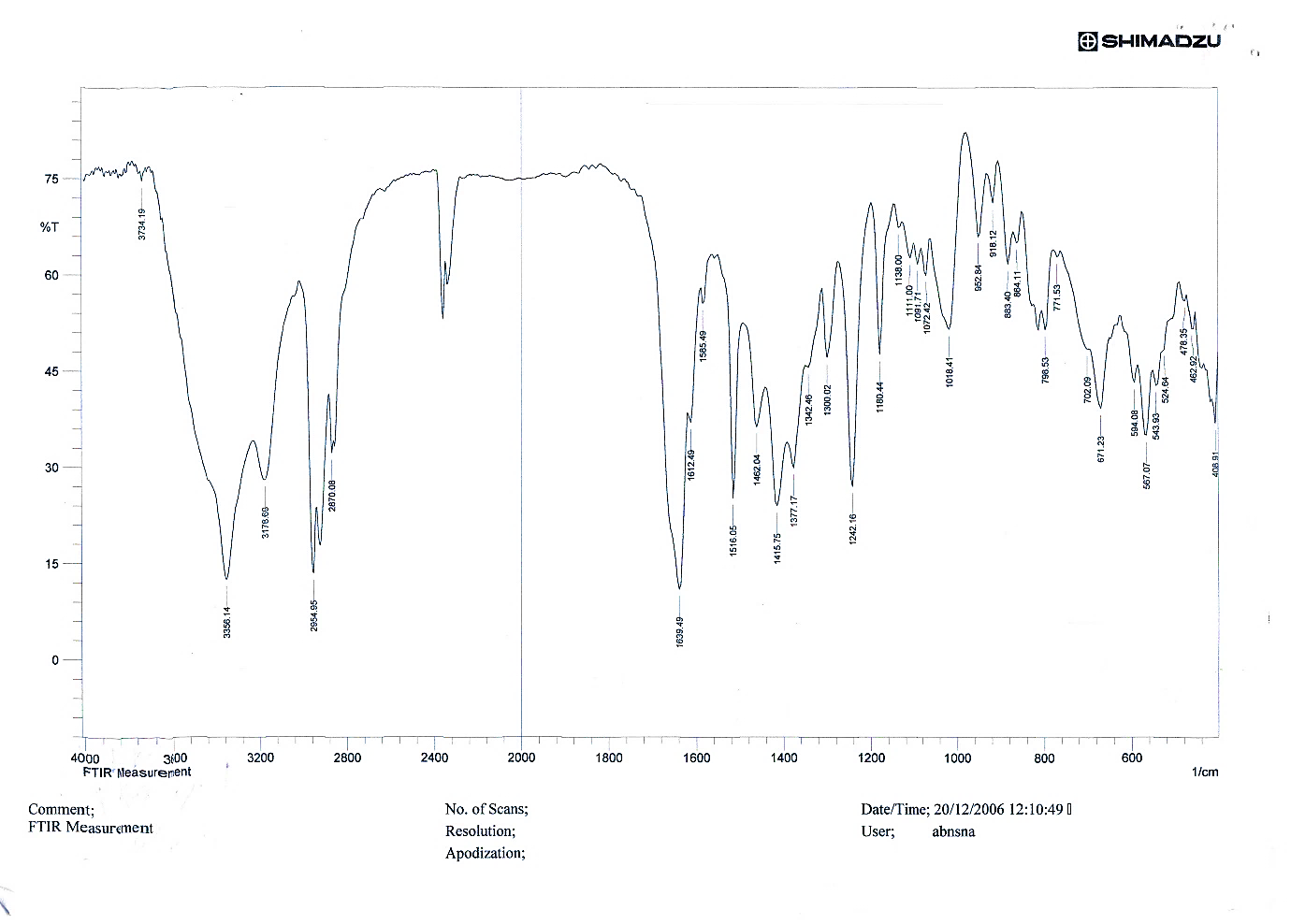


**Figure S8.** FTIR spectrum of complex **4**.


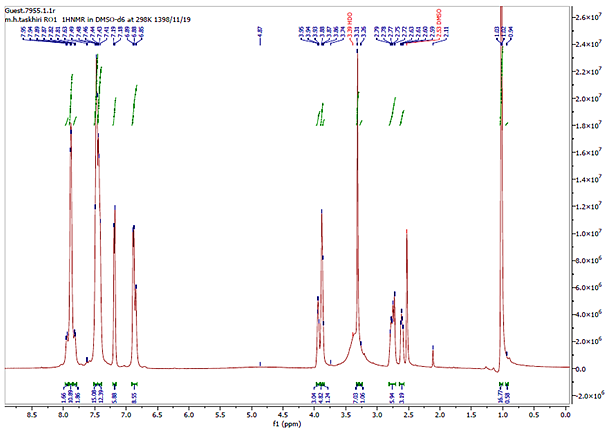


**Figure S9.** ^1^H NMR spectrum of complex **1**.


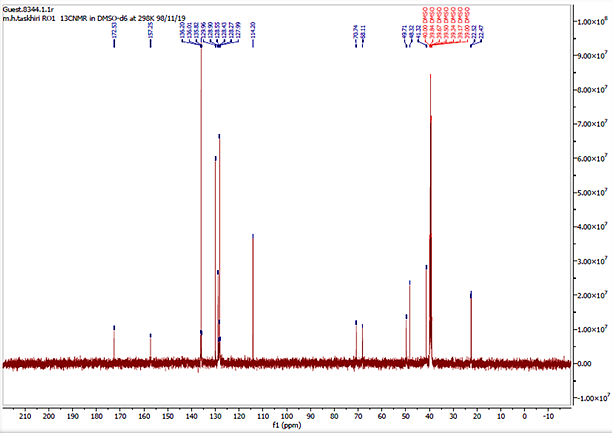


**Figure S10.** ^13^C NMR spectrum of complex **1**.


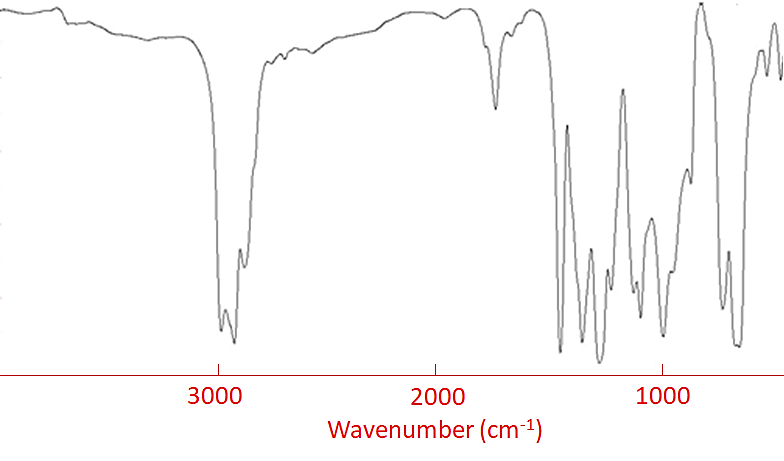


**Figure S11**. FTIR spectrum of PVC containing complex **1** after irradiation (300 h).

| 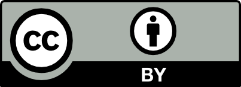 | © 2020 by the authors. Submitted for possible open access publication under the terms and conditions of the Creative Commons Attribution (CC BY) license (http://creativecommons.org/licenses/by/4.0/). |
| --- | --- |
